# PREDICTORS OF INTENTION TO WORK WITH OLDER ADULTS: THE CASE FOR STUDYING SOCIAL NORMS

**DOI:** 10.1093/geroni/igz038.2227

**Published:** 2019-11-08

**Authors:** Kirsten Graham

**Affiliations:** Colorado State University, Fort Collins, Colorado, United States

## Abstract

The expanding population of older adults, coupled with provider hesitance, is expected to result in a large service gap in the healthcare field. Research has largely focused on the impact of attitudes toward older adults and professional competency, with some recent explorations of social influences. There is currently no comprehensive measure that includes all of these areas. The present study outlines the development of the Working with Older Adults Scale (WOAS), which is grounded in the theory of planned behavior. Results indicated that the measure has an excellent factor structure and good internal reliability and construct validity. Intention to work with older adults was significantly predicted by attitudes, subjective norm, and perceived behavioral control, with subjective norm accounting for the greatest amount of variance. The WOAS offers new insights and ideas for future exploration of the service gap between older adults needs and professional availability across health service fields.

